# Self‐harm‐related mental imagery: A content analysis study of imagery reported by young people referred to mental health services

**DOI:** 10.1002/jcv2.12263

**Published:** 2024-10-30

**Authors:** Karima Susi, Anne Stewart, Rebecca Knowles Bevis, Keith Hawton

**Affiliations:** ^1^ Oxford Institute of Clinical Psychology Training and Research Isis Education Centre Warneford Hospital Oxford Health NHS Foundation Trust and University of Oxford Oxford UK; ^2^ Oxford Health NHS Foundation Trust Warneford Hospital Oxford UK; ^3^ Child and Adolescent Mental Health Services Leicestershire Partnership NHS Trust Leicester UK; ^4^ Step 4 Psychological Therapies Nottinghamshire Healthcare NHS Foundation Trust Nottingham UK; ^5^ Department of Psychiatry University of Oxford Warneford Hospital Oxford UK; ^6^ Department of Psychiatry Centre for Suicide Research University of Oxford Warneford Hospital Oxford UK

**Keywords:** self‐harm, self‐harm‐related mental imagery, suicide, young people

## Abstract

**Background:**

Growing evidence suggests that self‐harm‐related mental imagery is involved in the transition from self‐harm ideation to enactment. However, there has been little research on this important phenomenon in adolescent populations.

**Methods:**

Using an online questionnaire, the frequency, content and impact of self‐harm‐related mental imagery was investigated in a transdiagnostic clinical population of young people with recent self‐harm. Mood ratings were used to assess the impact of completing the questionnaire.

**Results:**

Fifty‐five young people aged 14–24 years old completed the study. Participants were mostly female (85.5%) and White (87.3%). All but one participant (98.2%) reported images related to self‐harm, with 53 (96.4%) reporting future‐oriented self‐harm images, 53 (96.4%) reporting past‐oriented images, and 52 (94.5%) reporting both. Imagery included imagining self‐harm and particularly dangerous acts (including suicide), specific methods, and the consequences of self‐harm for self and others. Past self‐harm‐related mental imagery was sometimes used to develop future‐oriented self‐harm‐related imagery planning, highlighting the influence of previous exposure to self‐harm. Most participants (*N* = 45; 88.2%) stated that significant self‐harm‐related mental imagery increased the likelihood they would self‐harm. Stimulation of mental imagery was most frequently reported to be related to dreams about self‐harm (*N* = 33; 60.0%), and exposure to self‐harm‐related content on social media (*N* = 32; 58.2%) and in fictional TV programmes (*N* = 30; 54.6%). There was no significant difference in participants' pre‐ and post‐questionnaire mood ratings.

**Conclusions:**

Self‐harm‐related mental imagery is commonly experienced by young people who self‐harm and may play a role in ideation‐to‐enactment of self‐harm. Asking about self‐harm‐related mental imagery can be done safely and could be considered for inclusion in routine clinical assessments. Self‐harm exposure and the origins of self‐harm‐related imagery, such as the links with past self‐harm and social media, as well as potential imagery‐based interventions for self‐harm, require further evaluation. A working model of self‐harm‐related mental imagery is presented.

## Graphical abstract

An online questionnaire examined the frequency and content of self‐harm‐related mental imagery in young people with recent self‐harm. All but one participant (98.2%) reported images related to self‐harm, and could include imagining self‐harm and dangerous acts (e.g. suicide), specific methods, and the consequences of self‐harm for self and others.

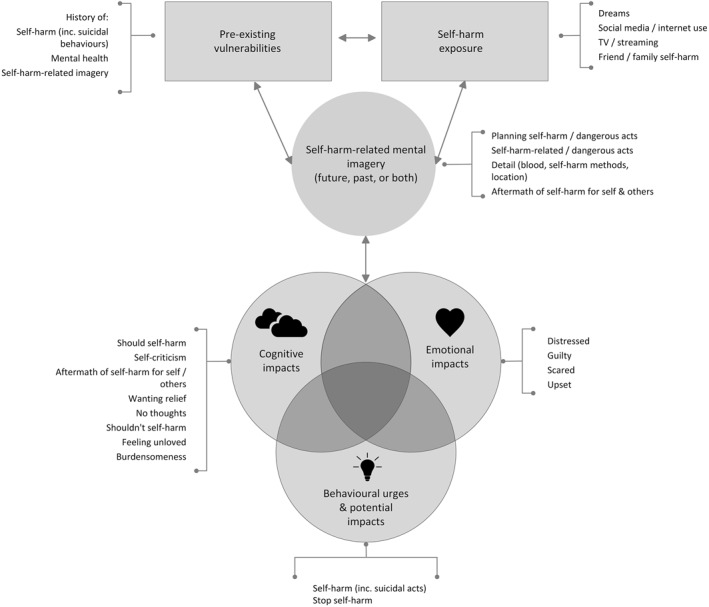

**Key points**

- Self‐harm and suicide‐related mental imagery has been implicated in self‐harm and suicidal behaviours and may play a role in ideation‐to‐enactment; however, there has been very little research in young people.
- This is one of the first studies to describe the frequency, content and impacts of self‐harm‐related imagery in a clinical sample of young people with recent self‐harm.
- Self‐harm‐related mental imagery was highly prevalent in the sample.
- Participants described a range of types of imagery, including of past acts and potential future acts of self‐harm.
- Asking about self‐harm‐related mental imagery did not have a significant impact on participants' mood.
- Clinicians should consider assessing self‐harm‐related mental imagery to guide risk assessment, formulation, and therapeutic interventions, in a sensitive manner that minimises the risk of harm.
- Further research is needed to understand the role self‐harm‐related mental imagery in self‐harm, the influence of exposure to previous self‐harm on both imagery and self‐harm, and the effectiveness of interventions targeting self‐harm‐related mental imagery in a diverse population of participants.

## INTRODUCTION

Self‐harm is defined as non‐fatal intentional self‐injury or self‐poisoning, irrespective of the motives involved, including suicidal intent (Hawton et al., 2003; National Institute for Health and Care Excellence [NICE], 2022). While some clinicians and researchers differentiate between suicidal and non‐suicidal self‐injury (NSSI), others find this unhelpful (Kapur et al., 2013), including the fact that NSSI excludes self‐poisoning. Rates of self‐harm are particularly high in young people, especially in females, including under‐16‐year‐olds (Geulayov et al., 2018). Self‐harm is associated with increased risk of suicide in both adults (Carroll et al., 2014), and young people (Hawton et al., 2020). In the UK, rates of suicide among children and adolescents have increased markedly since 2010 (Bould et al., 2019).

Identifying potential mechanisms contributing to self‐harm and suicidal thoughts in young people is important in order to develop more effective treatments and prevention strategies (Fox et al., 2020; Witt et al., 2021a, Witt et al., 2021b). There has been growing interest regarding the role of mental imagery in suicide and self‐harm. Mental imagery is a perceptual experience in the mind without an accompanying external stimulus, although often influenced by sensory stimuli experienced previously. Mental imagery can be multi‐sensory, but visual mental images are probably the most frequent and well‐studied. These are more emotionally activating than verbal thoughts (Holmes & Mathews, 2005) and are a strong predictor of future behaviour (Libby et al., 2007). There is a considerable body of research examining the brain representation of mental imagery, with studies demonstrating that visual mental images evoke similar activity patterns in the brain to visual perception (Pearson et al., 2015). The positive role of mental imagery in shaping behaviour has been applied to a variety of contexts, including sports and musical performance (Keller, 2012; Lindsay et al., 2023). In contrast, mental imagery has been implicated in several mental health disorders (Holmes & Mathews, 2010; Ji et al., 2019), such as post‐traumatic stress disorder (Ehlers & Clark, 2000), social anxiety (Chapman et al., 2020), suicidal behaviour (Holmes et al., 2007; Lawrence et al., 2021, 2023; Wesslau et al., 2015; Wetherall et al., 2018), and self‐harm (Dargan et al., 2016; Hasking, Di Simplicio, Mcevoy & Rees, 2017; McEvoy et al., 2017).

According to the integrated motivational–volitional model of suicidal behaviour, mental imagery can be a moderator of suicidal ideation‐to‐enactment, acting as a form of cognitive rehearsal for the behaviour, alongside other moderators such as availability of means of suicide, exposure to suicidal behaviour, capability for suicide, planning, impulsivity and previous suicidal behaviour (O’Connor & Kirtley, 2018). Depressed and suicidal individuals experiencing future‐oriented suicidal ‘flash‐forward’ mental imagery of future suicidal acts reported more severe suicidal ideation (Holmes et al., 2007). Psychiatrically‐hospitalised adolescents who reported suicidal mental imagery (approximately two‐thirds) had 2.4 greater odds of having made a suicide attempt than those who did not report any such mental imagery (Lawrence et al., 2021).

In a study of both suicidal and self‐harm‐related mental imagery in a Scottish sample of people aged 18–34 years, Wetherall et al. (2018) found that the presence of mental imagery distinguished those who had made a suicide attempt from those who had not. In a university student sample, the tendency to think in images moderated the relationship between negative affect and the odds of self‐harm (Hasking et al., 2017). McEvoy et al. (2017) found that more than 90% of adults experienced imagery‐based cognitions immediately prior to self‐harm, which most frequently included images of the self‐harm act. In a diary study of young adults, the frequencies of NSSI and mental imagery of this behaviour were correlated, with imagery almost always occurring before self‐harm (Cloos et al., 2020). A qualitative inquiry into self‐harm‐related imagery showed that young adults experienced both past‐oriented and future‐oriented imagery about self‐harm (i.e., past and future self‐harm acts), which could trigger self‐harm (Dargan et al., 2016). Whilst evaluating an imagery‐based intervention for self‐harm, a diary study by Di Simplicio et al. (2020) showed that 82% of young people aged 16–25‐years reported experiencing self‐harm‐related mental imagery prior to engaging in self‐harm. More recently, an ecological momentary assessment (EMA) study found that higher frequencies of NSSI mental imagery (flash‐forward to the actions of NSSI) predicted a greater urge to self‐harm and an increased likelihood of acting on an urge (Ji et al., 2024).

In summary, emerging evidence suggests that self‐harm‐related mental imagery may be involved in self‐harm ideation‐to‐enactment, with an increased risk of these behaviours amongst those who experience related mental imagery. However, most research has focused on suicidal imagery, with only a few studies exploring imagery related to non‐fatal self‐harm, most of which have been in adults and used quantitative methods (Lawrence et al., 2023). Few studies have investigated self‐harm‐related mental imagery in adolescent populations (Di Simplicio et al., 2020; Ji et al., 2024; Lawrence et al., 2021, 2023).

The aim of the current study was to investigate the phenomenology of self‐harm‐related mental imagery in a sample of young people who had recently self‐harmed, focussing on their imagery experiences and content, to address the following questions: (1) How common is mental imagery in a transdiagnostic clinical sample of young people? And (2) What types of mental imagery do young people experience? An additional exploratory objective was to provide preliminary data on the potential psychological impacts of this type of mental imagery.

## METHODS

### Participants and procedure

A convenience sampling approach was used to recruit eligible young people who had self‐harmed in the previous two months through clinicians in 21 National Health Service (NHS) community mental health services and one inpatient mental health service. The sample size was determined by the number of cases likely to provide a reasonable range of experiences. Participants were included in the study if they had self‐harmed in the previous two months, were aged between 14 and 24 years (as the age of adolescence is now considered to include those up to 24‐years of age (Sawyer et al., 2018) and self‐harm is common in the 18–24 year age group), were fluent in English, attending or had attended a mainstream school or college, had been assessed by an NHS child and adolescent or adult mental health service in Berkshire, Buckinghamshire, Leicestershire, or Oxfordshire, and were able to give informed consent (or assent and parental‐informed consent if <16 years). Individuals were excluded if they did not meet the above criteria, or had a diagnosis of psychosis, substance misuse, or were actively suicidal, due to risk concerns, the possibility of disturbing imagery being recalled and the potential influence of substances on images. Individuals were also excluded if they had a severe learning difficulty/disability or severe autism (defined as having not attended, or not being on roll to attend, a mainstream school because of their diagnosis), due to the likelihood of comprehension and communication difficulties precluding completion of the questionnaire.

Clinicians approached potential participants and shared a study information sheet. Those who expressed an interest in taking part were contacted by the first author (KS) to discuss the study and confirm eligibility. Eligible participants met with KS on Skype (as the study was conducted during the COVID‐19 pandemic) to give written informed consent (or assent and parental consent if <16 years), to clarify self‐harm and imagery definitions, and to complete the study questionnaire (which took 30–60 min). In total, 77 young people were referred for the study, but five declined to take part, eight were not eligible, eight did not respond to attempts to contact and one failed to attend agreed appointments. Therefore, 71% of those referred took part in the study.

### Measures and consultation

A self‐report questionnaire was devised by the authors. Consultation was held with service and non‐service users (aged 14–24 years) to examine the age appropriateness, comprehension, and importance of the questions. Following this feedback with stakeholders, changes were made to the wording and timeframe of some of the questions (e.g., reducing the timeframe for reporting previous self‐harm from three to two months as they felt this would increase the reliability of recall), and additional questions (e.g., about past self‐harm imagery) included. Data were collected via Qualtrics (2020). Piloting confirmed that two months was an appropriate time‐frame to use. Referring clinicians provided diagnostic information.

The questionnaire covered the following topics:

**Demographic information (age, gender, and ethnicity)**

**Information regarding self‐harm (frequency, type, reasons and emotion preceding self‐harm).** This was developed by the study authors as existing validated measures of self‐harm are less well‐suited to young people due to their length and the arbitrary distinction between non‐suicidal self‐harm and suicide attempts that is often made. The frequency of self‐harm was assessed by asking participants to estimate self‐harm frequency in the previous two months on a nine‐point Likert scale, which included the following anchor points: 0 (none), 1 (once), 2 (several days), 3 (on average 1–2 days per week), 4 (on average 3–4 days per week), 5 (on average 5–6 days per week), 6 (on average once every day), 7 (on average twice per day) and 8 (on average more than twice per day). These anchor points were chosen based on consultation and piloting and also helped young people estimate the total frequency of self‐harm in the last two months, which was the next question and was used to estimate the average self‐harm frequency. A two‐month timeframe was chosen for all measures following service user consultation and discussion regarding facilitating accurate recall. Information about the types of self‐harm carried out by participants in the previous two months was gathered using a free‐text response.

Reasons for the most recent episode of self‐harm (e.g., ‘I wanted to get relief from a terrible state of mind’) were taken from the list in Rodham et al.'s (2004) study and presented as tick‐box items. With this item and similar ones in the questionnaire, participants could select ‘other’ and provide a different response from those listed.

The Negative Affect subscale from the Positive and Negative Affect Schedule—Short Form (PANAS‐SF; Watson et al., 1988), was used as a state measure to assess the main emotion experienced immediately before self‐harm episodes in the previous two months (although participants could endorse emotions other than those on the PANAS‐SF). The PANAS‐SF has been used in self‐harm and imagery research and been found to have good psychometric properties in both adults and adolescents (Huebner & Dew, 1995), and across different timeframes (Watson et al., 1988). Internal consistency in this study was acceptable (*α* = 0.76).

**Self‐harm‐related mental imagery (frequency and content).** Participants were asked to estimate the frequency of future‐oriented and past‐oriented visual self‐harm‐related mental images occurring before self‐harm in the previous two months, on the same nine‐point Likert scale as used for self‐harm. The Likert scale was used to help young people estimate the total frequency of mental images in the previous two months, which was the next question. Previous research (Holmes et al., 2007) and stakeholder consultation highlighted the importance of making the distinction between past‐oriented and future‐oriented self‐harm. We did not define the timeframe of what ‘before self‐harm’ meant as pilot data indicated that imagery could span a large time‐frame but still contribute to subsequent self‐harm. Self‐harm‐related mental imagery was defined as any images considered by participants to be related to their self‐harm. Given the inherent difficulties in separating self‐harm‐related from suicide‐related mental imagery, self‐harm‐related mental imagery could include either type of act. KS completed a definition checklist with participants to ensure they understood what was meant by self‐harm and self‐harm‐related mental imagery before taking part.

The Mental Imagery Interview questions from previous imagery studies (i.e., Hales et al., 2011, Holmes et al., 2007, McEvoy et al., 2017) were adapted for this study, so that participants were asked to describe the content of their future and past‐oriented images relating to self‐harm experiences in the previous two months, as well as their most significant mental imagery and its cognitive, emotional and behavioural impacts, including whether their most significant imagery had made them more, less, or no more likely, to engage in self‐harm. We used the list of imagery types from the Mental Imagery Interview for participants to identify which subtype(s) of images they had experienced most. This was presented to participants after obtaining the free text imagery responses, to avoid influencing their free‐text responses.

**Exposure to self‐harm content.** Exposure to self‐harm‐related content in the previous two months was identified by asking participants to tick a list of possible exposure sources (e.g., self‐harm seen/heard via social media, friends or family). Participants could add other sources where relevant. They were asked to indicate whether each exposure source had led to self‐harm‐related mental imagery and/or self‐harm (i.e., Have any of the following resulted in you having images about self‐harm? Has self‐harm shown or discussed caused you to self‐harm?).

**Impact reduction and impact assessment measures.** The questionnaire ended by asking participants to describe positive images (if they had experienced these) or to imagine positive imagery (if they had not), as a mood elevation technique (Lloyd‐Richardson et al., 2015). An impact assessment tool (Rivlin et al., 2012) measured the impact on mood of asking about mental imagery, by asking participants to rate their mood (0 = worst; 100 = best) immediately before and after the study. Participants were asked about their experience of taking part and given a debrief sheet signposting them to sources of support if needed (e.g., Samaritans).

### Data analysis

Textual data arising from descriptions of self‐harm and self‐harm‐related mental imagery related to future, past, and most significant mental imagery were screened to establish the content and validity of responses. Any responses that were deemed invalid were removed and/or recoded under the correct category (e.g., if a participant responded to the past imagery question with something relating to imagining future self‐harm). A code book was developed by KS, AS, and KH to guide the data analysis by independently reviewing all the data and developing themes/subthemes. The authors met to compare, discuss, and resolve any inconsistencies. KS combined themes and coded the data using NVivo version 12. The new code book and coded data were shared with AS and KH to further refine and agree on coding as part of an iterative process. The frequency of coded themes and subthemes was usually calculated as a percentage of total valid responses. Two participants did not report any future‐oriented imagery, and one participant did not answer the question at all, so the percentage was calculated on the basis of a total of 52 participants. For the past‐oriented imagery data, two participants did not report any such imagery, so the percentage total was calculated out of 53 participants. For the overall subtypes of mental imagery responses, 54 responses were recorded. For various impacts of the most significant imagery, percentages were calculated based on the total number of valid responses (e.g., 45 people responded appropriately to cognitive impacts).

## RESULTS

### Participants

In total, 55 participants aged 14–24‐years were recruited over a period of nine months (08/2020–04/2021), two of whom were from an inpatient setting. Participants were mostly female (*N* = 47; 85.5% female) and White (*N* = 48; 87.3%).

### Pre‐existing vulnerabilities

#### Mental health diagnoses.

The two most common mental health diagnoses were depression (*N* = 31; 58.5%) and anxiety (*N* = 30; 56.6%). Some young people had more than one diagnosis. See Table 1 for further details.

**TABLE 1:** Demographic and self‐harm characteristics of participants (*N* = 55).

Characteristics	*N* (%)
Age	
Mean (SD) = 16.4 (1.8)	
14–17 years	47 (85.5%)
18–24 years	8 (14.5%)
Gender	
Female	47 (85.5%)
Male	5 (9.1%)
Other	2 (3.5%)
Prefer not to say	1 (1.8%)
Ethnicity	
White	48 (87.3%)
Asian	3 (5.7%)
Mixed	2 3.5%)
Black	1 (1.8%)
Other	1 (1.8%)
Diagnostic information *N = 53 referring clinicians	
Depression	31 (58.5%)
Anxiety	30 (56.6%)
Emerging personality disorder	11 (20.8%)
Social anxiety	10 (18.9%)
Autism spectrum disorder	9 (17.0%)
Post‐traumatic stress disorder/trauma	6 (11.3%)
Eating disorder	5 (9.4%)
Gender dysphoria	3 (5.7%)
Obsessive compulsive disorder	1 (1.9%)
Tourette's syndrome	1 (1.9%)
Self‐harm: Age of onset	
Mean (SD) = 12.6 (1.9)	
Range = 6–17 years	
Number of episodes in previous 2 months	
Mean (SD) = 13.5 (16.2)	
Range = 1–80	
Method(s) used in previous 2 months	
Cutting	54 (98.2%)
Overdose	19 (34.6%)
Headbanging	13 (23.6%)
Scratching	13 (23.6%)
Burning	8 (14.6%)
Hitting self	6 (10.9%)
Biting	5 (9.1%)
Pinching	5 (9.1%)
Pulling hair	5 (9.1%)
Hitting/punching walls	4 (7.3%)
Ligature	4 (7.3%)
Picking	3 (5.5%)
Bruising	2 (3.6%)
Trying to fall from height/break joints	2 (3.6%)
Rubbing	1 (1.8%)
Reasons for last self‐harm episode	
I wanted to get relief from a terrible state of mind	45 (81.8%)
I wanted to punish myself	33 (60.0%)
I wanted to die	25 (45.5%)
I wanted to show how desperate I was feeling	9 (16.4%)
I wanted to find out if someone really loved me	1 (1.8%)
I wanted to frighten someone	1 (1.8%)
I wanted to get some attention	1 (1.8%)
I wanted to get my own back on someone	0 (0.0%)
Other	9 (16.4%)
‐ ‘To feel something’	4 (7.3%)
‐ ‘Self‐disgust/hatred’	2 (3.6%)
‐ ‘Stop spiralling mindset’	2 (3.6%)
‐ ‘Out of addiction’	1 (1.8%)
Main emotion before self‐harm	
Distressed	19 (35.6%)
Upset	12 (21.8%)
Other	10 (18.2%)
‐ Anger	4 (7.3%)
‐ Numb/empty	3 (5.5%)
‐ Overwhelmed	2 (3.6%)
‐ Relief	1 (1.8%)
Guilty	7 (12.7%)
Nervous	3 (5.5%)
Ashamed	2 (3.6%)
Irritable	2 (3.6%)

#### Previous self‐harm.

Table 1 shows participants' reported information relating to self‐harm. The mean age of self‐harm onset was 12.6 years (SD = 1.9; range 6–17 years). On average, participants reported self‐harming 13.5 times (SD = 16.2) in the previous two months, with cutting reported as the most frequent method (*N* = 54; 98.2%), followed by overdosing (*N* = 19: 34.6%). The most frequently endorsed reasons for the last self‐harm episode related to internal motivations for self‐harming, such as wanting to get relief from a terrible state of mind (*N* = 45; 81.8%), wanting to punish myself (*N* = 33; 60.0%), and wanting to die (*N* = 25; 45.6%). Few participants endorsed externally‐motivated reasons, although some participants reported wanting to show others how desperate they were feeling (*N* = 9; 16.4%). Participants most frequently reported feeling distressed (*N* = 19; 35.6%) and upset (*N* = 12; 21.8%) before self‐harming.

#### Self‐harm exposure.

Table 2 shows the frequency of exposure to self‐harm‐related content, and whether such exposure led to mental imagery and/or self‐harm. The top three exposure sources were the Internet, social media, and fictional TV (all *N* = 40; 72.7%). Dreams about self‐harm (*N* = 33; 60.0%), exposure to self‐harm‐related content on social media (*N* = 32; 58.2%), and fictional self‐harm‐related content on TV (*N* = 30; 54.6%), were reported to generate mental imagery about self‐harm most frequently. Exposure to self‐harm images in dreams was most frequently reported to result in self‐harm (*N* = 28; 51.0%), followed by social media (*N* = 22; 40.0%), and exposure to friends' self‐harm (*N* = 20; 36.4%).

**TABLE 2:** Self‐harm‐related exposure sources in the previous two months and whether this led to mental imagery and/or self‐harm (*N* = 55).

Self‐harm‐related exposure source	Frequency of exposure in the previous two months *N* (%)	Frequency of mental imagery experienced in the previous two months following exposure *N* (%)	Frequency of self‐harm resulted after exposure to self‐harm content *N* (%)
Internet	40 (72.7%)	25 (45.5%)	17 (30.9%)
Social media	40 (72.7%)	32 (58.2%)	22 (40.0%)
Fictional TV (e.g., films, dramas)	40 (72.7%)	30 (54.6%)	14 (25.5%)
Dreams	33 (60.0%)	33 (60.0%)	28 (51.0%)
Friends self‐harming	33 (60.0%)	26 (47.3%)	20 (36.4%)
Non‐fictional TV (e.g., reality, news)	33 (60.0%)	21 (38.2%)	11 (20.0%)
Music	31 (56.4%)	21 (38.2%)	16 (29.1%)
Books	20 (36.4%)	15 (27.3%)	2 (3.6%)
Family self‐harming	15 (27.3%)	10 (18.2%)	10 (18.2%)
Music videos	15 (27.3%)	11 (20.0%)	4 (7.3%)
Magazines	7 (12.7%)	4 (7.3%)	2 (3.6%)
Radio	6 (10.9%)	3 (5.5%)	1 (1.8%)
Newspapers	4 (7.3%)	3 (5.5%)	2 (3.6%)
Other sources (i.e., school, diary, song lyrics, conversations about self‐harm)	8 (14.6%)	School: 6 (10.9%)	School: 3 (5.5%)
		Diary entries: 1 (1.8%)	Conversations about self‐harm: 1 (1.8%)
		Song lyrics written: 1 (1.8%)	
		Conversations about self‐harm: 1 (1.8%)	

### Frequency of self‐harm‐related mental imagery

Overall, 54 participants (98.2%) reported experiencing either future and/or past‐oriented self‐harm‐related mental imagery, with 52 (94.5%) reporting both. In the previous two months, 53 (96.4%) participants reported past‐oriented self‐harm‐related imagery. This had occurred on average: once (*N* = 3; 5.7%), several days (*N* = 15; 28.3%), 1–2 days per week (*N* = 7; 13.2%), 3–4 days per week (*N* = 13; 24.5%), 5–6 days per week (*N* = 4; 7.6%), once every day (*N* = 6; 11.3%), twice per day (*N* = 3; 5.7%), and more than twice per day (*N* = 2; 3.8%).

53 (96.4%) participants reported experiencing future‐oriented imagery before self‐harm. They said that this occurred on average: once (*N* = 2; 3.8%), several days (*N* = 12; 22.6%), 1–2 days per week (*N* = 18; 34%), 3–4 days per week (*N* = 6; 11.3%), 5–6 days per week (*N* = 5; 9.4%), twice per day (*N* = 6; 11.3%), and more than twice per day (*N* = 4; 7.5%).

### Themes of self‐harm‐related imagery

In reporting the self‐harm‐related mental imagery, we have calculated percentages based on those who actually reported experiencing imagery and provided valid responses. A summary of themes collected from participants' free‐text responses regarding the types of future‐ and past‐oriented mental imagery experienced is provided in Table 3 and discussed in the following sections. These include the planning of future self‐harm‐related and dangerous/suicidal acts, as well as the aftermath of such acts.

**TABLE 3:** Participants' descriptions of future and past self‐harm‐related mental imagery.

Theme	Future‐oriented self‐harm‐related imagery frequency *N* (%)	Future‐oriented illustrative quotes [subtheme, if applicable]	Past‐oriented self‐harm‐related imagery frequency *N* (%)	Past‐oriented illustrative quotes [subtheme, if applicable]
1. Self‐harm‐related acts	50 (96.2%)		44 (83.0%)	
1.1 self‐injurious acts	41 (78.6%)	‘*Seeing me cutting my arms*’ [cutting]	29 (54.7%)	*‘I have experienced images of times I had cut and the way the cuts looked, how much blood there was and where they were’* [cutting]
		*‘Seeing myself with rope around my neck and my face turning blue’* [dangerous acts]		*‘When I took my overdose”* [dangerous acts]
1.2 detail	29 (55.8%)	*‘Imagining blood coming out of the wounds if I cut my arms deep enough’* [blood & depth of self‐harm]	27 (50.9%)	*‘I can picture what I saw when I have previously self‐harmed, how my arm looks, how the blood runs, I can see myself sat in my room self harming in the past’* [injury detail; where on body; where they were]
		*‘…breaking shaving blades to get the razors and cut myself’* [accessing self‐harm objects]		*‘Images of different things I could use to self‐harm with’* [self‐harm objects]
		*‘Pictured self sat on bathroom floor cutting’* [where they were]		
		*‘Normally where specifically I would cut (so others wouldn't notice/easily be covered up) ’* [where they self‐injured]		
1.3 unspecified images of self‐harm	9 (17.3%)	*‘Planning, or visualising what/how I'm going to self‐harm’*	6 (11.3%)	*‘Images of me self harming in the past’*
2. Aftermath	35 (67.3%)		28 (52.8%)	
2.1 for self	28 (53.9%)	*‘What the cuts will look like after, the blood…’* [what skin looks like]	15 (28.3%)	*‘Seeing the after‐math (as in the cuts straight after, the blood etc) ’* [what self‐harm looks like]
		*‘Me hanging off a tree’* [dying]		*‘How to bandage a cut how to stop bleeding’* [cleaning it up]
		*‘How I was going to hide it, what I'd say if someone saw’* [how to hide self‐harm acts]		*‘Picturing the consequences like having to hide it and going to hospital’* [hiding self‐harm]
		*‘Feeling something different instead of the pain in my head the feeling of cutting’* [feeling different]		*‘Past self harm injuries, especially the deeper ones and the feeling of relief’* [relief]
2.2 for others	15 (28.9%)	*‘…sometimes if I feel suicidal, I will picture my own dead body in the bath and one of my family finding it’*	19 (35.9%)	*‘Family distressed, disappointed faces’*
				*‘The worried faces of my family and hospital staff’*
2.3 unspecified consequences of self‐harm	2 (3.9%)	*‘The consequences of it [self‐harm] ’*	4 (7.6%)	*‘The consequences of what happened after last time’*
3. Planning self‐harm‐related acts	20 (38.5%)	*‘Imagining how I want my own thighs to look also during the self harm (right before) ’*	–	–
		*‘I also try and push myself to hurt myself more than what I already have by picturing how bad it got. ’* [building on past self‐harm]		

*Note*: Future‐oriented images were calculated from *N* = 52 responses, and past‐oriented images were calculated were calculated from *N* = 53 responses.

#### Past‐oriented mental imagery related to acts of self‐harm.

Most participants (*N* = 53) reported mental images of past self‐harm‐related acts that had occurred before self‐harming in the previous two months (*N* = 44; 83.0%; see Table 3). These consisted of self‐injurious acts (*N* = 29; 54.7%), such as past cutting (*N* = 27; 50.9%), but a few participants also described imagery of burning (*N* = 2; 3.8%) or injuring their limbs in some way (*N* = 2; 3.8%). Some participants described imagery of more dangerous or suicidal self‐injurious acts they had completed in the past (*N* = 8; 15.1%), such as overdoses, ligaturing or attempting to hang themselves, and cutting their neck.

Of those reporting mental images of past self‐harm, half of them provided detailed descriptions of these images (*N* = 27; 50.9%). Typically, participants described an injury and associated blood in graphic detail (*N* = 17; 32.1%). Image descriptions included the site of self‐harm (e.g., arms, legs, neck; *N* = 11; 20.8%), their location (e.g., bedroom; *N* = 7; 13.2%), and method (e.g., razor blades, tablets, rope, glass; *N* = 6; 11.3%). The viewpoint or perspective taken was sometimes noted, such as seeing themselves cutting in the image (*N* = 3; 5.7%).

#### Future‐oriented mental imagery related to acts of self‐harm.

The majority of participants described their future self‐harm images (*N* = 52). As shown in Table 3, the majority of these images related to future self‐harm acts (*N* = 50; 96.2%), which most often involved cutting (*N* = 30; 57.7%), which was usually congruent with their most frequently described self‐harm method in the previous two months. Twenty‐three participants (44.2%) described images related to methods incongruent with their usual acts, such as dangerous/suicidal acts, which included taking overdoses, jumping from a high place, ligaturing, deeply cutting their neck or other parts of the body, and completing suicide. Some participants (*N* = 6; 11.5%) described seeing themselves from an observer perspective in their mental images. Two participants (3.8%) reported imagining the feeling of cutting in their description of imagery, with one specifically stating imagining the physical pain they would experience.

Over half of participants (*N* = 29; 55.8%) reported specific details of their images. These included graphic descriptions of blood coming out of a wound or the depth of self‐injury (*N* = 17; 32.7%), objects they would use (e.g., blades, pills, rope, sharp objects; *N* = 10; 19.2%), the site of self‐harm on the body (e.g., arms, legs; *N* = 7; 13.5%), and their imagined location, such as a bathroom, a bridge, hospital, or train station (*N* = 9; 17.3%).

Mental imagery involving the planning of future self‐harm‐related acts was described by 38.5% of participants (*N* = 20); for example, how and where they would cut and what this would look like. A small number of participants (*N* = 4; 7.7%) described imagery which involved building on past self‐harm, such as self‐harming more severely or hiding it more effectively.

#### Imagery related to the aftermath of self‐harm‐related acts.

For past‐oriented imagery descriptions, over half of participants (*N* = 28; 52.8%) reported imagery relating to the aftermath of previous self‐harm‐related episodes. Most commonly this related to the consequences for others (*N* = 19; 35.9%), remembering how others reacted negatively to finding out about their self‐harm such as parents being upset, disappointed, or disgusted, or being shouted at or lectured, or the concerned faces of family and staff. The aftermath of self‐harm for themselves was also reported as a focus for imagery by 28.3% of participants (*N* = 15). This included what the wound looked like afterwards (*N* = 7; 13.2%) and having to clean up or hide the self‐harm (both. *N* = 4; 7.5%).

For future‐oriented imagery, approximately two‐thirds of participants (*N* = 35; 67.3%) described experiencing images relating to the aftermath of engaging in future self‐harm‐related acts (Table 3). These included the consequences of self‐harm for themselves (*N* = 28; 53.9%), such as what their skin would look like (*N* = 17; 32.7%) or of dying (*N* = 7; 13.5%). Imagery also related to the consequences for others (*N* = 15; 28.9%), particularly how they would react and what they would see.

### Overall subtypes of mental imagery

The most frequently endorsed type of self‐harm‐related mental imagery selected from a predetermined list of imagery subtypes (presented after the participants free text responses) related to a time they had tried to self‐harm in the past (*N* = 43; 79.6%), followed by a distressing event that happened to them in the past (*N* = 41; 75.9%), and planning or preparing to harm themselves in the future (*N* = 41; 75.9%) (see Table 4).

**TABLE 4:** Overall subtypes of self‐harm‐related mental imagery experienced in the two months before self‐harm (*N* = 54).

Type of mental imagery experienced	Frequency of mental imagery *N* (%)
A time you tried to self‐harm in the past	43 (79.6%)
A distressing event that happened to you in the past	41 (75.9%)
Planning/preparing to harm yourself in the future	41 (75.9%)
What might happen to you if you self‐harmed	36 (66.6%)
What might happen to other people/how they would react	32 (59.3%)
A difficult thing you wanted to escape from	30 (55.6%)
Something that you heard/saw in person	25 (46.3%)
Something that you heard/saw online or on social media	21 (38.9%)
Things that were fleeting/unclear	11 (20.4%)
Something that made you feel safe/better	10 (18.5%)

### Impacts of self‐harm‐related mental imagery

Participants' reported impacts related to their most significant self‐harm‐related images, which could be future, past or both (Table 5).

**TABLE 5:** Cognitive and emotional impacts and behavioural urges reported for most significant self‐harm‐related mental imagery.

Impacts	Illustrative quotes [what type(s) of significant imagery experienced]	*N* (%)
Cognitive (*N* = 45)		
Should self‐harm	*‘That I needed and wanted to do it [self‐harm] ’* [future and past mental images of cutting]	19 (42.2%)
Self‐criticism	*‘if I don't get there [self‐harm until it matches mental image] I'm pathetic & I'm just faking everything’* [future and past mental images of cutting]	6 (13.3%)
Aftermath of self‐harm for self and others	*‘How it would make me feel, how to hide it from others around me if I was to do it’.* [future and past images of cutting]	5 (11.1%)
	*‘They [images of cutting in the past] made me upset since I don't want to hurt others around me’*	
Wanting relief	*‘I want it again [cutting] because the kind of feeling of emotional release’.* [future images of cutting]	5 (11.1%)
No thoughts	*‘didn't really have thoughts but images of me doing it’* [future and past images of cutting]	4 (8.9%)
Shouldn't self‐harm	*‘I thought about how I didn't want to do it again but then I also did want to’* [future images of cutting]	4 (8.9%)
Burdensomeness	*‘Like I was never meant to be born into this world, that I don't deserve to live anymore’* [future mental images of cutting and suicidal acts]	3 (6.7%)
Confusion	*‘I felt confused of why I was in the position I was and I wish I could have done something about it but it also makes me want to hurt myself so bad again so I have deeper scars’* [past images of painful emotions felt when cutting]	3 (6.7%)
Fearful of self	*‘It made me think that I should be scared of myself’* [future and past images of cutting]	3 (6.7%)
Feeling unloved	*‘I thought that I am unloved by anyone and nobody will care if I die’.* [future and past mental images of cutting]	3 (6.7%)
Inevitability of self‐harm	*I felt like I didn't have any control about it. I thought it [taking an overdose] was inevitable. ‘* [future mental images of taking an overdose]	3 (6.7%)
Self‐harm very real	*‘I thought it [cutting mental image] was very vivid, there were a lot of details, and I thought it was very concerning that it made me feel so proud but also that it was very realistic’* [future and past mental images of cutting]	3 (6.7%)
Emotional (*N* = 51)		
Distressed	*‘It made me feel quite distressed to be able to think like that’.* [past images of cutting]	15 (29.4%)
Guilty	*‘Guilt, I know no one around me would want me to do it [self‐harm] so I feel bad that I still do’.* [self‐harm‐related image from the past]	15 (29.4%)
Scared	‘*Quite scared as knowing that*'*s what my head wants whether I want to badly self harm or not If I get them thoughts then my impulsivity always gets what it want*’ [images of future suicide‐related acts]	14 (27.5%)
Upset	*‘They [mental images of past self‐harm] made me upset since I don't want to hurt others around me but it made me feel secure that I have something there that I could do if I need to. I.E cutting’.*	11 (21.6%)
Numb/lack of emotion	*‘It [mental images of taking an overdose in the future] made me feel numb…’*	6 (11.8%)
Relieved	*‘Wanted me to have that cut on my arm so I could feel a relief’* [mental images of cutting in the past]	6 (11.8%)
Ashamed	*‘I was ashamed I was having the images’* [cutting or overdosing in the future and past]	5 (9.8%)
Sad	*‘It made me feel sad…’* [mental images of cutting in the future and past]	5 (9.8%)
Behavioural urges (*N* = 51)		
Self‐harm	*‘All I wanted to do was hurt myself because I felt like I needed to like I needed to be punished for how I make myself feel. I'd imagine me doing it [self‐harm] and then I would do it’.* [mental images of self‐harming in the future]	37 (72.5%)
Dangerous acts	*‘Slit my throat and die… I had a very strong urge to do it’.* [mental images of future and past]	13 (25.5%)
Stop self‐harming	*‘Stop self harming completely or a lot more’* [mental images of cutting in the past]	6 (11.8%)

*Note*: Only themes with >2 participants for cognitive impacts, and ≥5 participants for emotional and behavioural impacts, are shown.

**Cognitive impacts:** 19 participants (42.2%) reported a belief that they should self‐harm in response to mental imagery. Others (*N* = 6; 13.3%) experienced self‐critical thoughts after having images. Following imagery, 11.1% of participants (*N* = 5) thought about the possible aftermath of self‐harm‐related acts on themselves and others (e.g., having to hide self‐harm, upsetting others). 11.1% (*N* = 5) also thought about how they wanted relief from their emotional pain in response to imagery. Other cognitive themes are presented in Table 5.

**Emotional impacts:** A wide range of emotions were described by participants in response to their most significant images (Table 5). Participants described feeling distressed or guilty (both *N* = 15; 29.4%), scared (*N* = 14; 27.5%), and upset (*N* = 11; 21.6%).

**Behavioural impacts:** Behavioural urges reported by participants in relation to images (Table 5) most often included wanting to engage in self‐harm (*N* = 37; 72.5%). Some participants described how mental images made them want to self‐harm more frequently and severely. When specifically asked about the effect their most significant self‐harm‐related mental images had on their urge to self‐harm, 45 out of 51 participants (88.2%) reported that they were more likely to self‐harm. Five participants (9.8%) reported no effect on their self‐harm, and one participant reported that they were less likely to self‐harm in the context of recalling images of the impact of past self‐harm on loved ones.

Urges to engage in dangerous acts following significant mental images were reported by 25.5% of participants (*N* = 13), which included taking overdoses and other potentially suicidal acts, such as ligaturing. Only 11.8% (*N* = 6) reported having urges to stop self‐harm, but often these occurred alongside urges to self‐harm.

### Impact of completing the questionnaire

There was no statistically significant difference between participants' mood ratings before and after completing the survey (pre: mean (out of 100) = 51.6; SD = 16.7; post: mean = 50.6; SD = 17.3); [*t* (54) = 0.96, *p* = 0.34]), indicating that being asked about self‐harm and imagery did not significantly lower participants' mood. Additionally, only one person endorsed, in their free‐text response, a link between conversations about self‐harm and frequency of self‐harm after this exposure (Table 2).

## DISCUSSION

### Main findings

Overall, 98.1% (*N* = 54) of young people with recent self‐harm reported experiencing future and/or past‐oriented self‐harm‐related mental imagery, with 96.4% reporting future‐oriented imagery, 96.4% reporting past‐oriented imagery, and 94.5% reporting both. This finding indicates that self‐harm‐related imagery was highly prevalent in this transdiagnostic clinical sample of young people and is consistent with other research reporting that a high percentage of adults and young people experience self‐harm‐related mental imagery before self‐harm (Di Simplicio et al., 2020; McEvoy et al., 2017).

The content of both future and past‐oriented images most often included self‐harm‐related acts, which could include dangerous acts that might result in death, detailed descriptions of such acts (e.g., including blood), as well as the aftermath of these acts for the self and others. Self‐harm‐related imagery was typically congruent with participants' main self‐harm methods (i.e., cutting and overdoses), but not always as some participants also reported imagery related to completing more dangerous acts, including suicide. Some participants reported imagery related to accessing self‐harm objects, which highlights the importance of asking about access to imagined means during clinical assessments. These findings are in keeping with those of previous studies, mostly conducted with adult samples of people with self‐harm (Cloos et al., 2020; Dargan et al., 2016; Di Simplicio et al., 2020; Hasking et al., 2017; Lawrence et al., 2013, 2021; McEvoy et al., 2017; Wesslau et al., 2015). The qualitative data in this study regarding the content of self‐harm‐related imagery provide additional detail consistent with the recent EMA study by Ji et al., 2024, which found that 59.3% of individuals aged 17–24 (with a history of self‐harm) reported an increased urge to self‐harm following self‐harm‐related imagery. Of potential importance is the fact that future‐oriented images also included planning self‐harm‐related acts, such as being competitive with oneself to extend on past self‐harm‐related acts. This highlights the importance for clinicians to have full knowledge of previous self‐harm when assessing the potential implications of future self‐harm‐related mental imagery.

Whilst the study precludes inferring causality, self‐harm‐related mental images were reported by participants as facilitating their self‐harm. The majority of participants (88.2%) reported being more likely to self‐harm following exposure to their most significant self‐harm‐related mental images, which is in keeping with recent research showing that self‐harm‐related mental imagery may be associated with increased risk for self‐harm behaviour (Cloos et al., 2020; Ji et al., 2024; Lawrence et al., 2023). In view of previous work showing an increased risk of suicide in adolescent psychiatric inpatients who experienced suicide‐related mental imagery (Lawrence et al., 2021) it is notable that some participants reported thoughts of taking overdoses, ligaturing, or of suicide. Participants reported that self‐harm images could increase thoughts that they should self‐harm, as well inducing self‐criticism, and thinking about the relief that self‐harm would bring. Similar findings were reported by McEvoy et al., 2017 in a study of undergraduates. Emotional impacts of imagery often matched the reasons given for self‐harm, such as feeling distressed and wanting relief from a terrible state of mind.

In contrast, there was also some evidence that imagery could be protective and deter self‐harm, where some participants reported urges to stop self‐harming following imagery. This is consistent with previous literature indicating that imagery regarding the impact of self‐harm on others could reduce the urge to self‐harm (McEvoy et al., 2017). The recent EMA study by Ji et al., 2024 found that 14.1% of participants reported a decreased urge to self‐harm following self‐harm‐related imagery, although the specific content of the imagery was not specified.

Participants reported that exposure to self‐harm‐related content via dreams, fictional TV, social media, and friend's self‐harm‐related behaviours were the most frequent stimuli for self‐harm‐related mental imagery and/or self‐harm. Whilst other research has shown the impacts of social media and the Internet (Susi et al., 2023), TV and related streaming platforms such as Netflix (Arendt et al., 2019), and friends' self‐harm (Hasking et al., 2015), the role of dreams (an item included in the current study following service‐user consultation) has received less attention.

Importantly, pre‐ and post‐questionnaire mood ratings indicated that asking about self‐harm‐related mental imagery did not lead to a significant reduction in participants' mood. Inclusion of positive imagery stimulation at the end of the questionnaire may have helped in this regard. Moreover, only one participant endorsed a link between conversations about self‐harm and frequency of self‐harm after this exposure (although this was a ‘write‐in’ response).

**FIGURE 1:**
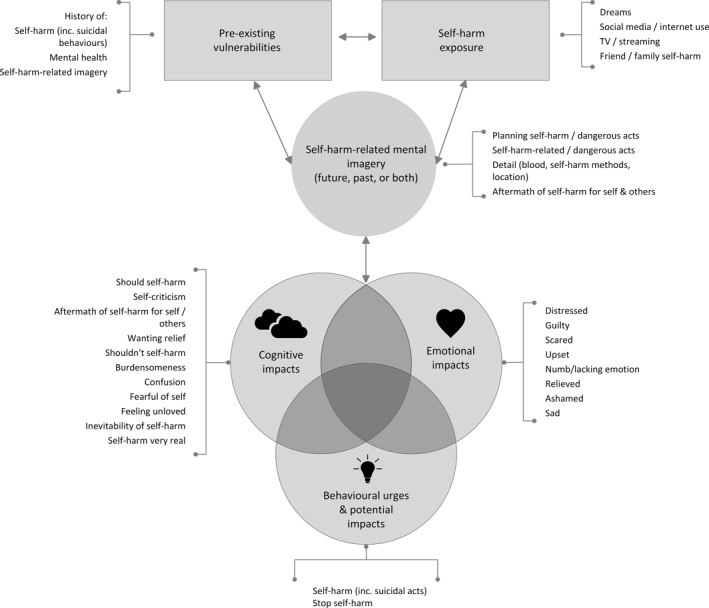
Working model of self‐harm‐related mental imagery to assist with future research and clinical assessment.

Overall, the findings support current theories in the field, and suggest that self‐harm‐related‐mental imagery may be a volitional moderator involved in the transition from self‐harm and suicidal ideation to related behaviours in young people (O’Connor & Kirtley, 2018). Investigations of self‐harm‐related mental imagery in young people are in their infancy. In order to assist conceptualisation of the nature of imagery and its potential impacts we have developed Figure 1 using the findings obtained from the study. This includes cognitive and emotional themes related to acquired capability for a suicidal act, perceived burdensomeness and thwarted belonging, consistent with Joiner's interpersonal theory of suicide (2005).

### Strengths and limitations of study

One of the study's strengths is that it is among the first to explore self‐harm‐related mental imagery in young people who had recently self‐harmed, including a detailed content analysis of both future and past‐oriented imagery which has highlighted the links between them. The study also explores possible links between self‐harm exposure sources (e.g., social media, TV, friends, family and dreams) and how this may have contributed to some young people experiencing mental imagery and/or subsequently engaging in self‐harm.

The study is cross‐sectional so cannot determine causality or directionality. It is thus not clear from the findings whether mental imagery contributes to self‐harm or is a by‐product of self‐harm, or both. Most participants were White and female, so the extent to which the findings can be generalised is limited. Also, the size of the sample precluded examination of differences in relation to age. The main method of self‐harm was cutting, which may not be representative of individuals who present to other services (e.g., hospital) or who do not seek help (Geulayov et al., 2018). In addition, in order to include all types of self‐harm we used a non‐validated measure of this behaviour. Retrospective self‐reporting may be subject to bias through overgeneralised recall of affect, imagery, and their impacts (Pile et al., 2021; Williams et al., 1996), although accuracy is often better for emotional memories (Buchanan, 2007). Also, participants' responses suggested that imagery was highly salient for this sample. However, it is possible that participants may have been more likely to recall imagery when the self‐harm intention was acted on. Young people who experience self‐harm‐related imagery may have been more likely to volunteer for the study than those who did not, although most participants reported never having been asked about mental imagery prior to taking part in the study. Moreover, the proportion of the sample who reported self‐harm‐related mental imagery is similar to that found in other studies (Di Simplicio et al., 2020; McEvoy et al., 2017). We did not recruit individuals who had not self‐harmed, as ethical concerns precluded this. Therefore, it is unclear whether, or to what extent, those who do not self‐harm experience self‐harmed‐related mental imagery. However, in Dargan et al.’s (2016) qualitative study of adults who self‐injured, several participants reported first experiencing imagery after they had started self‐harming, highlighting the influence of previous exposure to self‐harm.

Ideally, we would have like to have explored the association between specific elements of imagery and different types of self‐harm behaviour. However, the sample was too small for this analysis. A further limitation is the broad nature of the content analysis. However, we have conducted an in‐depth qualitative analysis with a subgroup of the participants which will be reported elsewhere.

### Implications for clinical practice

Given the prevalence of imagery related to self‐harm, it may be helpful for clinicians conducting clinical assessments to routinely ask about the presence and content of future and past‐oriented images (and the links between them), self‐harm exposure sources, and reported impacts of self‐harm‐related imagery (particularly on their well‐being and behaviour), and to use this information in formulation, and also safety planning, where appropriate (see Figure 1 for a framework to guide questioning). Given the lack of research and guidelines in this area, it is unlikely that clinicians routinely assess self‐harm‐related mental imagery in practice. This may be due to misconceptions that asking about imagery may increase risk, which was not the case in this study, at least in terms of changes in mood ratings. However, asking about self‐harm imagery needs to be completed sensitively and have a clear purpose, such as assessing and managing risk and delivering interventions. Although there was no significant drop in mood for participants following the completion of the questionnaire, it is possible that discussion about self‐harm imagery not done cautiously could trigger further self‐harm imagery, and actual self‐harm in some individuals. Further work evaluating the effectiveness of such clinical approaches is needed.

### Implications for future research

Future work in the area should investigate both self‐harm‐related and suicidal imagery and the influence of self‐harm exposure sources (e.g., dreams, social media, TV/streaming services, friends and family), particularly considering that self‐harm is an important risk factor for suicide in young people (Hawton et al., 2020). Longitudinal designs could be used to provide an in‐depth understanding of the role self‐harm‐related mental imagery plays in the pathway to self‐harm and suicide. Further exploration of other imagined sensations (e.g., pain, smells, tastes) and auditory experiences (e.g., sounds/conversations) would also be a topic for future exploration as this was beyond the scope of the present study. Future studies should also aim to recruit more diverse samples, particularly in terms of gender and ethnicity and also to ensure good representation across the spectrum of socio‐economic deprivation.

Innovative imagery‐based interventions for young people who self‐harm (Di Simplicio et al., 2020), where the distressing components of imagery are targeted through evidence‐based imagery techniques, may prove fruitful avenues for further exploration (e.g., rescripting images, eye movement desensitisation and reprocessing therapy (EMDR), or imagining the consequences of self‐harm on loved ones).

## CONCLUSION

Self‐harm‐related mental imagery was highly prevalent in a transdiagnostic clinical sample of young people who recently self‐harmed. Such imagery was reported by most participants to increase the likelihood of self‐harm, and therefore may be involved in the transition from self‐harm thoughts to related behaviours. Further research investigating self‐harm‐related mental imagery in young people is needed, using methodologies that are less reliant on retrospective recall, such as experience sampling. Asking about self‐harm‐related mental imagery did not lead to a significant reduction in pre‐ and post‐questionnaire mood ratings, but further research is warranted. Clinicians should consider asking young people about the nature, sources, and impacts of self‐harm‐related mental images to aid clinical risk assessment, formulation, and therapeutic interventions, but caution needs to be taken to minimise any risk associated with asking about self‐harm images.

## AUTHOR CONTRIBUTIONS

**Karima Susi**: Conceptualization, data curation, formal analysis, investigation, methodology, project administration, writing – original draft, writing – review & editing. **Anne Stewart**: Conceptualization, formal analysis, methodology, supervision, writing – original draft, writing – review & editing. **Rebecca Knowles Bevis**: Conceptualization, methodology, supervision, writing – review & editing. **Keith Hawton**: Conceptualization, formal analysis, methodology, supervision, writing – original draft, writing – review & editing.

## CONFLICT OF INTEREST STATEMENT

KH is a member of the National Suicide Prevention Strategy for England Advisory Group. All other authors declare that they have no competing or potential conflicts of interest.

## ETHICAL CONSIDERATIONS

National Health Service (NHS) Health Research Authority (HRA) ethical approval was given by West Midlands Black Country Research Ethics Committee (ref: 20/WM/0148).

## Data Availability

The data that support the findings of this study are available on request from the corresponding author. The data are not publicly available due to privacy and ethical restrictions.
